# Dysfunction of the Glymphatic System as a Potential Mechanism of Perioperative Neurocognitive Disorders

**DOI:** 10.3389/fnagi.2021.659457

**Published:** 2021-06-07

**Authors:** Xuli Ren, Shan Liu, Chuang Lian, Haixia Li, Kai Li, Longyun Li, Guoqing Zhao

**Affiliations:** ^1^Department of Anaesthesiology, China-Japan Union Hospital of Jilin University, Changchun, China; ^2^Department of Neurology, First Affiliated Hospital of Jilin University, Changchun, China; ^3^Department of Anaesthesiology, Jilin City People's Hospital, Jilin, China; ^4^Jilin University, Changchun, China

**Keywords:** glymphatic system, postoperative complications, postoperative neuropathy, postoperative cognitive dysfunction, perioperative neurocognitive disorders

## Abstract

Perioperative neurocognitive disorder (PND) frequently occurs in the elderly as a severe postoperative complication and is characterized by a decline in cognitive function that impairs memory, attention, and other cognitive domains. Currently, the exact pathogenic mechanism of PND is multifaceted and remains unclear. The glymphatic system is a newly discovered glial-dependent perivascular network that subserves a pseudo-lymphatic function in the brain. Recent studies have highlighted the significant role of the glymphatic system in the removal of harmful metabolites in the brain. Dysfunction of the glymphatic system can reduce metabolic waste removal, leading to neuroinflammation and neurological disorders. We speculate that there is a causal relationship between the glymphatic system and symptomatic progression in PND. This paper reviews the current literature on the glymphatic system and some perioperative factors to discuss the role of the glymphatic system in PND.

## Introduction

Perioperative neurocognitive disorder (PND), which encompasses delirium and postoperative cognitive dysfunction, commonly occurs in the elderly after anesthesia and surgery. Previously, all forms of postoperative cognitive impairments were called “postoperative cognitive dysfunction,” but more recently, the use of “perioperative neurocognitive disorder” is recommended as an overarching term for cognitive impairment identified in the preoperative or postoperative period (Evered et al., 2018a). This change aligns PND with the phenotypically similar neurocognitive diagnoses listed in the Diagnostic and Statistical Manual of Mental Disorders, version 5, such as Alzheimer's disease (AD) (Evered et al., 2018a). Furthermore, PND has emerged as a significant global public health issue that leads to more extended in-hospital stays, higher hospitalization costs, and higher mortality rates (Yang and Terrando, 2019). The risk factors for PND are multifaceted, which might be related to anesthetic management, tissue damage, surgery duration, psychological stress, and genetic susceptibilities (Subramaniyan and Terrando, 2019; Eckenhoff et al., 2020). As the elderly population continues to increase, the number of cases of PND will continue to rise. The incrementally growing prevalence forces researchers to explore the mechanisms underlying the pathogenesis of PND and to seek optimal prevention and treatment measures (Evered and Silbert, 2018; Berger et al., 2019).

Preclinical and clinical studies support the notion that neuroinflammation plays a significant role in the pathogenesis of PND (Nathan, 2019; Yang et al., 2019, 2020). Trauma experienced during surgery triggers the release of endogenous factors known as damage-associated molecular patterns that can activate immune cells such as neutrophils and monocytes, promote pro-inflammatory cytokines, and subsequently cause systemic inflammation (Huber-Lang et al., 2018). Uncontrolled systemic inflammation is associated with a compromised blood-brain barrier (BBB). BBB impairment promotes immune cell movement and pro-inflammatory cytokines from the blood into the brain parenchyma, triggering neuroinflammation and ultimately leading to postoperative cognitive impairment (Abrahamov et al., 2017; Yang et al., 2017, 2020). Microglia, the resident immune cells of the central nervous system (CNS), perform “immune surveillance,” and survey their assigned brain regions (Kettenmann et al., 2011). In addition, with prolonged systemic inflammation, microglia start to develop an activated phenotype characterized by an increase in pro-inflammatory mediators, such as interferon-γ, interleukin (IL)-1β, tumor necrosis factor (TNF)-α, and reactive oxygen species (Liu L. R. t al., 2020). Pro-inflammatory factors released by microglia such as IL-1α and TNF contribute to the subsequent activation of astrocytes and further promote neuroinflammation (Liddelow et al., 2017; Liu L. R. t al., 2020). Complement system activation is another essential inflammatory response that is activated by surgically triggered damaged-associated molecular patterns. For example, the deposition of the C-reactive protein, a biomarker for delirium, can activate and regulate the classical complement pathway, thereby contributing to dysregulated inflammation. Blocking the complement cascade (e.g., complement C3 gene deficiency and selective complement inhibitor for complement C3) improves neuroinflammation and functional outcomes of PND (Alawieh et al., 2018) also suggests the contribution of neuroinflammation in PND.

Several studies have shown that the pathological mechanism of PND is similar to that of AD (Evered et al., 2016; Gerlach and Chaney, 2018). It was also demonstrated that a significant accumulation of amyloid-β (Aβ) and tau in the brain parenchyma after anesthesia and surgery (Terrando et al., 2011). Moreover, increased Aβ and tau in the cerebrospinal fluid (CSF) have been reported in patients after surgery (Evered et al., 2018b), which are risk factors for PND (Xie Z. et al., 2013; Evered et al., 2016; Cunningham et al., 2019). The accumulation of Aβ and tau induces neuroinflammation, leading to glial activation, pro-inflammatory factor release, and neuronal damage (Calsolaro and Edison, 2016).

The glymphatic system is a recently discovered waste removal system that utilizes a unique perivascular channel system formed by astrocytes (Iliff et al., 2012; Benveniste et al., 2017; Zhang C. et al., 2018). Although the glymphatic system contributes to the delivery of nutrients, specifically glucose, its most influential and recognized function is to clear extracellular metabolites and waste products from the parenchyma into the CSF (Abbott et al., 2018; Nedergaard, 2013; Simon and Iliff, 2016). Since its discovery, researchers have proposed that waste or protein aggregates induced by glymphatic dysfunction are associated with neurodegenerative diseases, including AD (Tarasoff-Conway et al., 2015; Harrison et al., 2020; Nedergaard and Goldman, 2020). Given the characteristics of the glymphatic system and the complexity of possible changes during the perioperative stage, we herein speculate a causal relationship between the glymphatic system and symptomatic progression in PND. Glymphatic dysfunction in the perioperative period, which leads to waste accumulation, could trigger or exacerbate neuroinflammation and eventually lead to cognitive dysfunction. Through this review, we discuss the function and driving mechanism of the glymphatic system, outline the current evidence to illustrate the impact of anesthesia and surgery on the glymphatic system, and emphasize the viewpoint that glymphatic dysfunction is involved in the pathogenesis of PND.

## Glymphatic Pathway

**Figure 1:**
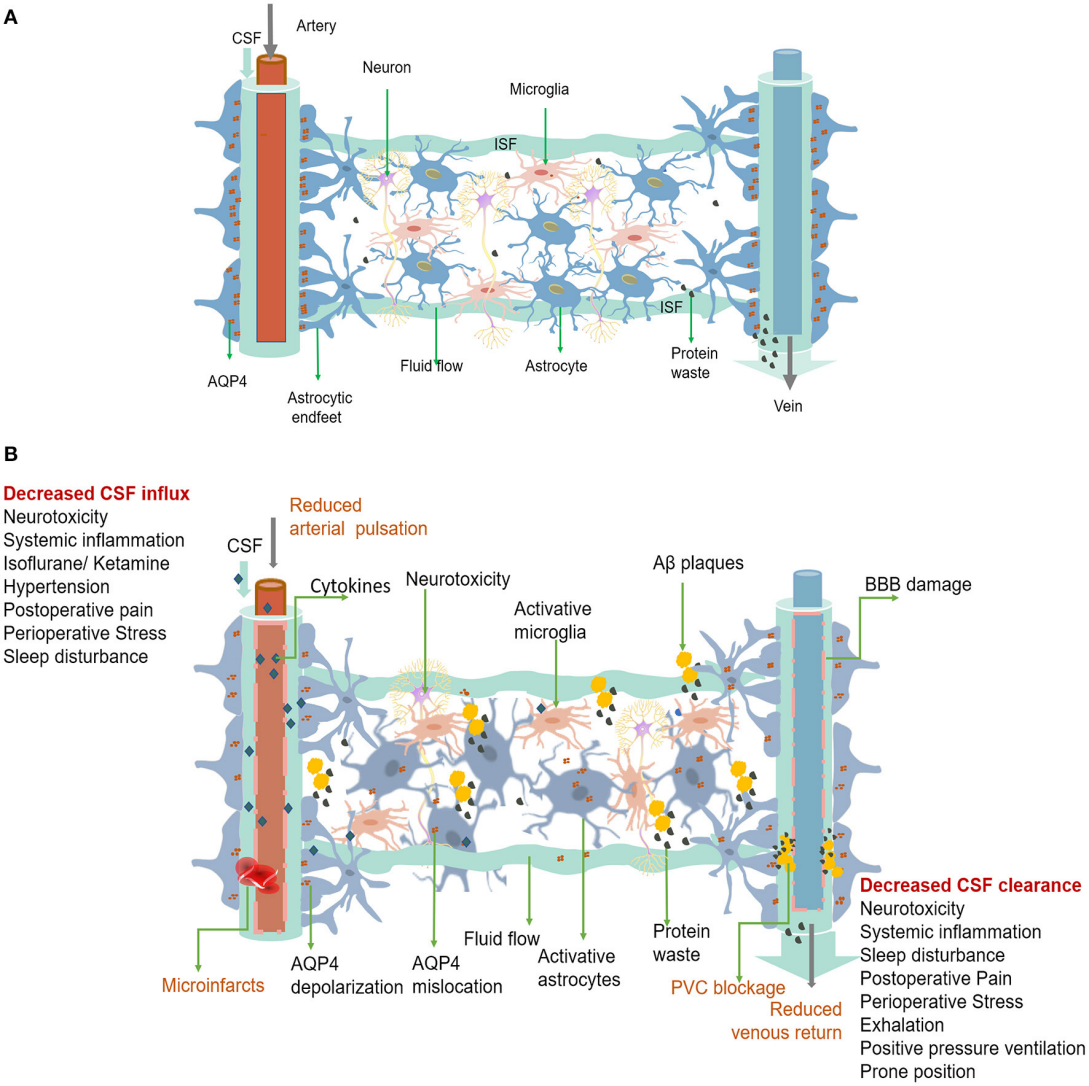
**(A)** The glymphatic system is a perivascular channel formed by astrocyte end-feet throughout the brain. CSF enters the brain parenchyma through the periarterial space, exchanges with ISF, and finally exits through the perivenous space. Rapid exchange of CSF within ISF is facilitated by AQP4, which is anchored to the astrocytic end-feet. Interstitial solutes, including protein waste, are drained from the brain with CSF through the perivenous space and via the meninges and cervical lymphatics. **(B)** Dysfunction of the perioperative glymphatic system. Perioperative anesthetic drugs can cause hemodynamic changes that reduce arterial pulsation mechanism change and decrease the inflow of the glymphatic system. Surgically induced systemic inflammation can cause blood-brain barrier opening and glymphatic system damage, leading to neuroinflammation and decreased waste clearance. Both the entry of peripheral inflammatory substances and the accumulation of protein wastes in the brain, such as Aβ accumulation and folding, can activate astrocytes and microglia and trigger neuroinflammation. Neuroinflammation can worsen the damage to the function and structure of the glymphatic system. Forceful expiration, positive pressure ventilation, and prone position can cause a decrease in venous return, leading to a decrease in CSF clearance. Pain, preoperative stress, and sleep disturbances can affect both CSF inflow and clearance. Glymphatic dysfunction can lead to a more significant accumulation of protein and waste products, which can trigger neuroinflammation and lead to PND. PVC, Perivascular space.

The glymphatic system is a perivascular network that subserves a pseudo-lymphatic function throughout the brain (Figure 1). Iliff et al. named this brain-wide fluid transport pathway the glial-associated lymphatic system or glymphatic system due to its dependence on glial water flux and the lymphatic function of the brain (Iliff et al., 2012). As the macroscopic waste clearance system of the CNS, the glymphatic system's fundamental role is to eliminate soluble proteins and metabolites, including Aβ and tau, based on the evidence that 40%−80% of neurotoxic compounds can be cleared from the CNS via this system (Iliff et al., 2012). The neurotoxic compound clearance process within the glymphatic system is described as a 3-step serial process (Benveniste et al., 2019b). First, CSF is continuously transported from the subarachnoid space and Virchow-Robin space to the peri-arterial spaces in a bulk-flow driven manner; subsequently, CSF is propelled from the peri-arterial compartment into the interstitial fluid (ISF) space. The convection and mixing of CSF and ISF are facilitated by aquaporin 4 (AQP4) in the dense and complex brain parenchyma. Ultimately, CSF-ISF fluid mixes with interstitial waste solutes and is subsequently transported to the perivenous space, from the meningeal lymphatic vessels to the lymphatic vessels and circulatory system (Plog and Nedergaard, 2018).

AQP4 is required to maintain the glymphatic function and is abundantly expressed in the end-feet of astrocytes that surround arteries and veins. It is also anchored to the astrocyte membrane by the carboxyl terminus of α-syntrophin. Syntrophin-dependent AQP4 mediates the bidirectional transport of water across the brain-blood interface (Amiry-Moghaddam et al., 2003). As a bidirectional channel, the effects of AQP4 on glymphatic function may be diverse and require further investigation. Although the role of AQP4 in promoting ISF clearance is debated, a large body of evidence suggests that it facilitates CSF movement from perivascular spaces into the interstitial space and activates flushing of ISF (Smith et al., 2017). Studies on different strains of AQP4-knockout mice have suggested that AQP4 is necessary for the rapid movement of CSF from the perivascular space into the ISF and through the brain (Mestre et al., 2018a). Moreover, AQP4 reduces the resistance to CSF movement from the periarterial space into the interstitium and subsequently from the interstitium into the perivenous space (Groothuis et al., 2007). The flow of CSF via the glymphatic pathway is an essential part of the CNS that eliminates metabolic waste products, such as Aβ and lactic acid, from deep inside the brain and delivers nutrients, such as glucose, or therapeutic drugs to the brain parenchyma. This CSF circulation differs from typical lymphatic vessels and acts as a pseudo-lymphatic drainage within the CNS. Therefore, more recently, alternative CSF circulation in the brain has been proposed as a glymphatic circulation (Benveniste et al., 2019b).

## The Driving Mechanism of Glymphatic System

### Arterial Vascular Pulsation

Unlike other peripheral organs, limited tissue compliance of the rigid skull facilitates the propagation of arterial pressure pulsations throughout the brain, resulting in measurable pulsatile blood flow and venous outflow in the microvasculature (Shi et al., 2020). This cardiac pulsation along the entire vascular bed is believed to be the most critical physical mechanism for glymphatic propulsion, facilitating CSF movement in the glymphatic system (Kyrtsos and Baras, 2015; Kiviniemi et al., 2016; Hablitz et al., 2020). Cardiovascular pulsation is also the fastest driving mechanism of the glymphatic system, originating in the basal peri-arterial spaces around the Willis's circle and extending centrifugally toward the cerebral cortex (Kiviniemi et al., 2016). The dynamic source is mainly due to the oscillation of the arterial wall caused by the heartbeat, which produces a net flow consistent with the blood flow direction and the same frequency as the cardiac cycle (Iliff et al., 2013; Kiviniemi et al., 2016). Each cardiac cycle involves a fast expansion of the artery wall, followed by a slow contraction. The bulk flow rate of CSF, calculated by CSF viscosity and the shape, cross-sectional area, and length of the vessels, is proportional to the hydraulic pressure drop along the vessels and inversely proportional to the hydraulic resistance (Thomas, 2019). The combined effects of diffusion and advection also play a role in clearing solutes from the brain (Thomas, 2019). The arterial wall pressure generates the hydraulic pressure of the CSF, which allows CSF to be delivered to the brain via the glymphatic system, a process in which the contractility of the vessel wall or the slow wave of vasomotor tension also plays an important role (Kiviniemi et al., 2016). Any reduction in vessel wall contractibility or slow contraction in vasomotor tone increases backflow and decreases net flow in the glymphatic system (Mestre et al., 2018b). CSF flow may be mechanically regulated by pressure differences, which may be a neurophysiological regulatory mechanism. For example, astrocyte calcium activity has been shown to propagate along blood vessels as waveforms and regulate water permeability and ion exchange in the perivascular space (Rangroo Thrane et al., 2013). Additionally, intracranial pressure and heart rate are related to cardiac pulsation and affect glymphatic function. Increased intracranial pressure (ICP) decreases the mean arterial pressure and impairs the function of the glymphatic system (Chen et al., 2018), while glymphatic influx correlates negatively with heart rate (Hablitz et al., 2019).

Research has shown that the glymphatic cross-section is a non-concentric elliptical space outside the vessel, which provides the least hydraulic resistance (Tithof et al., 2019). This cross-section model is quite different from the usual circular annulus models, which assume that the small arteries are located in the center of the space (Mestre et al., 2018b; Thomas, 2019). Moreover, Mestre et al. have shown that the glymphatic system infrastructure around pial arteries is 10 times larger *in vivo* than previous estimates after fixation based on electron microscope images (Tithof et al., 2019). As such, the glymphatic system offers much less viscous resistance to CSF flow than previously thought. These results also demonstrate the importance of further *in vivo* studies on the glymphatic system.

### Respiratory Forces

Advances in imaging and computational techniques have demonstrated that the respiratory mechanism is another important process that dominates the perivenous space (Kiviniemi et al., 2016). Although the respiratory force is not the primary driver of CSF flow, it is a modulating factor that supplements cardiac pulsation (Dreha-Kulaczewski et al., 2015). Respiratory dynamics are related to extreme low-frequency vasomotor oscillations during perivenous fluid movements (Kiviniemi et al., 2016). A study using magnetic resonance spin labeling in humans showed that CSF movement was enhanced during deep inhalation and was inhibited during deep exhalation (Yamada et al., 2013). A possible explanation is that forced inspiration causes blood to be drawn from the brain through the veins into the thorax, resulting in a decrease in intracerebral venous pressure, an expansion of the perivenous space, and an increase in CSF outflow from the perivenous space into the brain. Moreover, small but rapid CSF movement was observed during breath-holding, which is thought to reflect cardiac pulsations (Yamada et al., 2013). Alternatively, exhalation reduces venous outflow from the brain in a low-pressure venous drainage system (Kiviniemi et al., 2016). Additionally, respiration produces lower-frequency ICP oscillations and hydraulic pressure changes (Dreha-Kulaczewski et al., 2015).

### Circadian Regulation

**Figure 2:**
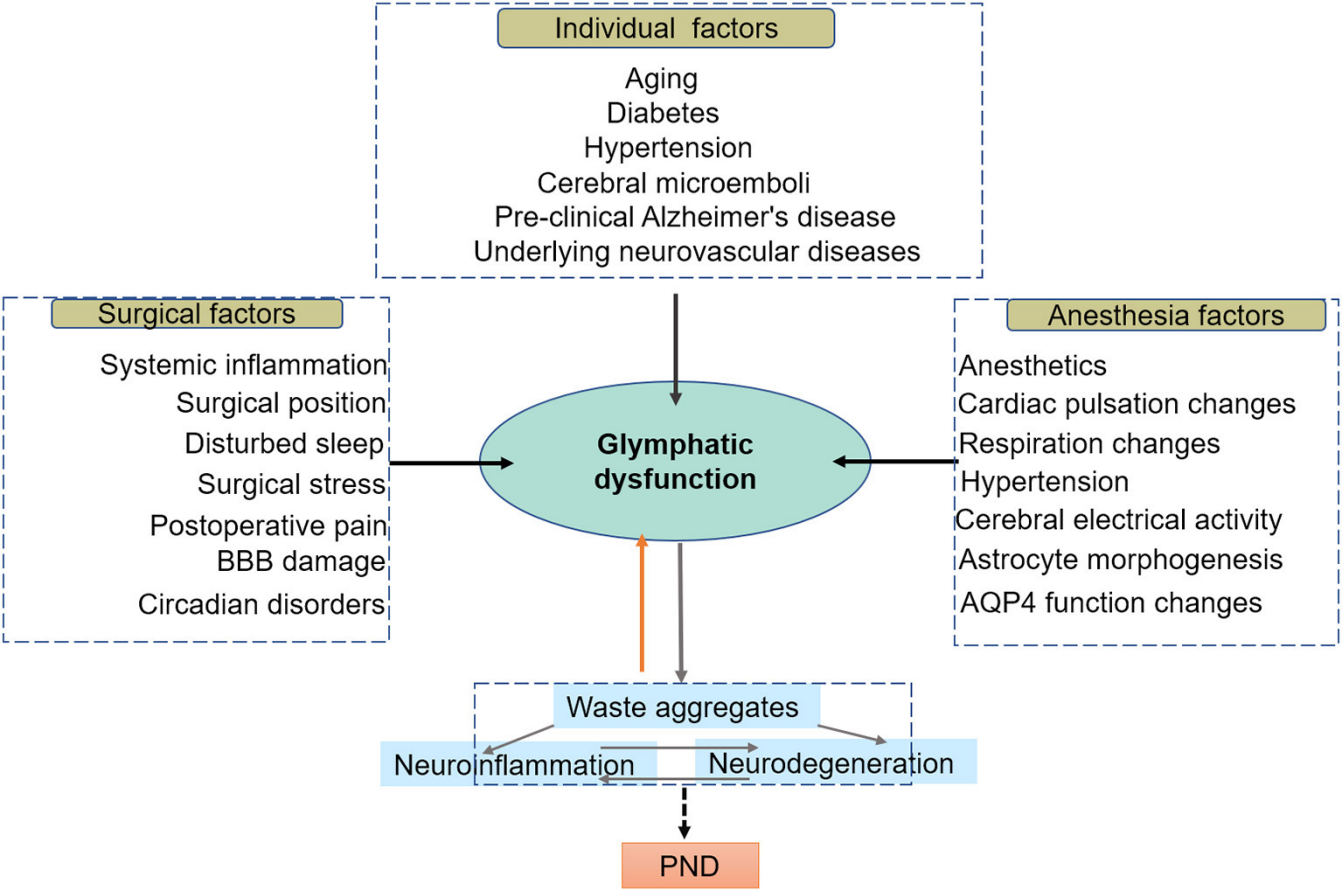
In this model, the glymphatic system resides at the intersection of a broad scope of perioperative risk factors, which share an association with diminished brain waste clearance. Individual factors are preexisting impairments in glymphatic function prior to surgery; anesthetic and surgical factors are associated with a dramatic decline in perioperative glymphatic function, compromising the glymphatic system and exacerbating the progression of preexisting disease. Glymphatic system dysfunction, in turn, contributes to protein aggregation and misfolding, leading to neuroinflammation, neurodegeneration, and ultimately PND.

Sleep is essential for the maintenance of healthy brain functions. Hence, early research suggests that glymphatic clearance is mainly associated with the sleep-wake cycle (Xie L. et al., 2013). Studies in rodents have shown that CSF absorption in the perivascular space and ISF flushing increase during sleep (Xie L. et al., 2013). Glymphatic activity is regulated by circadian regulation, with a daily rhythm that peaks at midday when mice are mostly likely to sleep (Hablitz et al., 2020). Similarly, glymphatic activity was more significant at night than during the day in patients administered with gadolinium as a CSF tracer to evaluate glymphatic function (Eide and Ringstad, 2019). The mechanism by which the circadian rhythm of the brain regulates glymphatic activity remains unclear. Circadian neural activities can be detected with an electroencephalogram (EEG) device (Dijk, 1999). Overnight EEG has shown that delta (1–4 Hz) power and sigma (12–15 Hz) power are high during non-rapid eye movement (NREM) sleep and low during rapid eye movement (REM) sleep. In contrast, beta (23–30 Hz) power is higher in REM sleep than in NREM sleep, except for an artifactual peak during cycle 2. EEG activity that resembles sleep or wakefulness tightly correlates to the glymphatic influx (Hablitz et al., 2019). The brain's waste clearance mainly occurs during the NREM stage, while the CSF influx was strongly suppressed during wakefulness (Hablitz et al., 2019, 2020). Sigma power during NREM fluctuates reciprocally with delta, becoming high when delta is low and low when delta is high (Campbell, 2009). It has been demonstrated that either high delta rhythm or slow oscillation (<1 Hz), which is a characteristic of NREM sleep, is positively correlated with the glymphatic function, while beta power that resembles wakefulness is negatively correlated with the glymphatic function (Hablitz et al., 2019). The underlying relationships between EEG power and glymphatic activity remains unclear. A recent study has revealed a coherent pattern of oscillating electrophysiological, hemodynamic, and CSF dynamics during NREM sleep (Fultz et al., 2019). These results suggest that neural activity may affect the function of the glymphatic system through hemodynamic circadian rhythms (Figure 2). Lactate concentration in the brain also closely corresponds to EEG activity and is considered the best metabolic biomarker of the sleep-wake cycle (Lundgaard et al., 2017). Lactate concentration decreases during the transition from wakefulness to sleep, leading to an expansion of the extracellular space and a decrease in intra-tissue resistance results, contributing to faster CSF influx into the interstitium and finally solute efflux.

## Glymphatic Function Affected by Anesthetics

Currently, most animal experiments on glymphatic function are performed by applying anesthetic drugs to simulate the sleep state. The effect of anesthetics cannot be ignored when studying the effect of sleep on glymphatic function. Currently, limited research has been conducted to investigate the effects of anesthetics or sedatives on the rodent glymphatic system. A study demonstrated that inhaled isoflurane (2–2.5%) inhibited CSF circulation and waste clearance in the brain (Gakuba et al., 2018). This result indicates that inhaled isoflurane inhibited glymphatic function, especially at a high dose (3%) (Gakuba et al., 2018). This research also showed that ketamine, a common intravenous anesthetic, also inhibited glymphatic activity at an intraperitoneal dose of 150 mg·kg^−1^ in mouse models (Gakuba et al., 2018). The inhibitory effect of ketamine on glymphatic function could be reversed using a combination of ketamine and 10 mg·kg^−1^ of xylazine (Gakuba et al., 2018). However, Xie et al. previously found that ketamine (100 mg·kg^−1^) combined with xylazine (20 mg·kg^−1^) significantly increased the influx of the CSF tracer in all mice analyzed (Xie L. et al., 2013). The opposite effects of the two different anesthetic regimens require further study. One possible explanation is whether ketamine inhibits or increases lymphatic inflow, depending on the dose of ketamine and its combination with xylazine, an α_2_-adrenergic agonist and an analog of clonidine used as a sedative and analgesic in animals. Xylazine increases glymphatic CSF influx and may remedy the impairment of ketamine use in the glymphatic system (Gakuba et al., 2018).

Dexmedetomidine (DEXM) is another α_2_-adrenergic agonist widely used for anesthesia or sedation in clinics and has also been shown to enhance glymphatic function in animal research (Lilius et al., 2019a). Recently, Ozturk et al. reported that DEXM supplemented with low-dose isoflurane increased glymphatic transport compared to that of isoflurane only (Ozturk et al., 2021). DEXM has also been shown to increase the delivery of intrathecally administered drugs, such as oxycodone and naloxone, by increasing glymphatic flow (Lilius et al., 2019b).

Propofol is a widely used intravenous anesthetic in humans with anti-inflammatory (Ren et al., 2014) and neuroprotective properties (Miller et al., 2018). Recent studies have found that propofol can increase glymphatic activity (Gakuba et al., 2018). Pentobarbital has also been shown to significantly increase glymphatic function in animal models (Hablitz et al., 2019), although benzodiazepines have mainly been replaced by human anesthesia. Other anesthetics used in animal anesthesia such as α-chloralose and avertin inhibit CSF tracer influx in the glymphatic pathway (Hablitz et al., 2019). Therefore, glymphatic function is affected by the dose, type, and combination of anesthetics. Although the exact mechanisms underlying these preclinical experiments remain unclear, they may provide valuable information for studying the functional effects of the glymphatic system during clinical anesthesia (Table 1).

**Table 1:** The effect of verified factors on glymphatic system.

**Classification**	**Perioperative factors**	**Glymphatic function**	**Classification**	**Perioperative factors**	**Glymphatic function**
Anesthetic factors	Neurotoxicity	Impair (Rangroo Thrane et al., 2013)	Cardiovascular factors	Acute hypertension	Impair (Mestre et al., 2018b)
				Chronic hypertension	Impair (Mortensen et al., 2019; Koundal et al., 2020)
	Isoflurane (2–2.5%)	Inhibit (2–2.5%) (Gakuba et al., 2018)		Heart rate	Negative correlation (Hablitz et al., 2019)
	Sevoflurane	Enhance (2.5%) (Gao et al., 2019)		Dobutamine	Enhance (Iliff et al., 2013; Yamada et al., 2013)
	Dexmedetomidine	Enhance (20 mg.kg^−1^; ip) (Hablitz et al., 2019; Lilius et al., 2019a)		Norepinephrine	Inhibit (Jessen et al., 2015)
	Xylazine	Enhance (Hablitz et al., 2019)	Surgical factors	Neuroinflammation	Impair (Yu et al., 2019)
	Ketamine	Inhibit (150 mg.kg^−1^)(with xylazine, 10 mg.kg^−1^) (Gakuba et al., 2018)		Position	lateral > supine > prone (Lee et al., 2015)
		Enhance (100 mg.kg^−1^)(with xylazine 20 mg.kg^−1^) (Hablitz et al., 2019)		Sleep disturb	Impair (Nedergaard and Goldman, 2020).
	Propofol	Enhance (Gakuba et al., 2018)		BBB damage	Impair (Meng et al., 2019; Yu et al., 2019)
	Pentobarbital	Enhance (60 mg.kg^−1^; ip) (Hablitz et al., 2019)		Postoperative Pain	Impair (Chouchou et al., 2014)
	α-chloralose	Inhibit (80 mg.kg^−1^; ip) (xylazine Hablitz et al., 2019)		Perioperative Stress	Impair (Wei et al., 2019)
	Avertin	Inhibit (120 mg.kg^−1^; ip) (Hablitz et al., 2019)	Individual factors	Preclinical stage of AD	Impair (Masters et al., 2015)
Arouse stage	Wake	Inhibit (Xie L. et al., 2013; Eide and Ringstad, 2019)		Neurovascular diseases	Impair (Riba-Llena et al., 2018)
	Sleep	Enhance (Xie L. et al., 2013; Alawieh et al., 2018)		Aging	Impair (Iliff et al., 2013)
	EEG	Beta power inhibit (Hablitz et al., 2019)		Apo E gene	Impair (Mentis et al., 2020)
		Delta power enhance (Hablitz et al., 2019)		AQP4 gene	Impair (Hubbard et al., 2018).
Respiration	Free breathing	N/A		Chronic hypertension	Impair (Mestre et al., 2018b; Riba-Llena et al., 2018)
	Deep inhalation	Enhanced (Dreha-Kulaczewski et al., 2015)		Diabetes	Impair (Zhang et al., 2014; Jiang et al., 2017)
	Mechanical ventilation	N/A	Other factors	Intracranial pressure	Increase will impair (Dreha-Kulaczewski et al., 2015)

Interestingly, these animal studies on glymphatic dysfunction agree with those of clinical studies on PND. Anesthetics are a risk factor for postoperative cognitive decline or neuropathological changes (Schenning et al., 2016). It has been confirmed that inhaled anesthetics, such as isoflurane or sevoflurane, have more chance of causing postoperative cognitive dysfunction (Ologunde and Ma, 2011; Hu et al., 2014), whereas some intravenous anesthetics, such as DEXM and propofol, cause slight harm or even improve perioperative cognitive function (Miller et al., 2018). Anesthetics that have a significant inhibitory effect on the glymphatic system are more likely to cause perioperative cognitive impairment, whereas some anesthetics that enhance glymphatic function are less likely to cause cognitive impairment. The effects of ketamine on the glymphatic system and postoperative cognition have been controversial (Morrison et al., 2018). DEXM enhances glymphatic function and has also been shown to prevent postoperative delirium and cognitive dysfunction caused by inhaled anesthetics in elderly patients (Zhang et al., 2018). Therefore, anesthetics may affect postoperative brain function by affecting the glymphatic function. Currently, the mechanisms underlying the effects of anesthetics on glymphatic function remain unclear. Further clinical research is necessary to determine the optimal variety, dose, and combination of anesthetics and drugs to reduce glymphatic function impairment during anesthesia.

## The Underlying Mechanism of Altered Glymphatic Function During Intraoperative Anesthesia Management

The mechanisms that regulate glymphatic function under normal physiological conditions include arterial pulsatility, respiration, and neural activity. However, these mechanisms may be disrupted during anesthesia, including general anesthesia and spinal anesthesia (Scott, 2018). During clinical anesthesia, the heart rate, blood pressure, and arterial pulsation are significantly changed, and spontaneous negative pressure respiration is replaced with positive pressure ventilation. The arousal mechanism is also significantly suppressed during general anesthesia (Schiff, 2020). Anesthesia-induced pathological processes, such as the breakdown of glymphatic CSF-ISF exchange, have been linked to disease initiation and progression. As a result, the impact of anesthesia on glymphatic function needs to be considered.

### Cardiac Pulsation Mechanism Change

The sympathetic, stellate ganglion, and subcutaneous sympathetic nerves are all suppressed by most anesthesia types, including general, neuraxial, epidural, and lumbar anesthesia. Anesthesia suppresses blood pressure and heart rate by causing dose-dependent vasodilation or vasoplegia. Hypotension occurs due to anesthesia-induced reduction in heart rate, vasodilation, reduced afterload, and circulating volume. Furthermore, a lower heart rate has a strong positive association with glymphatic influx (Hablitz et al., 2019). Currently, there is no research on the relationship between hypotension and glymphatic activity. Mestre et al. found that the jerky and irregular reversal of microparticle flow caused by increased arteriole stiffness and pulsation amplitude in distal vessels during hypertension reduced perivascular pump dynamics and decreased net CSF flow and waste clearance in the perivascular space (Mestre et al., 2018b). Additionally, CSF production is related to sympathetic or adrenergic receptor activity. Increased sympathetic tone inhibits carbonic anhydrase associated with the choroidal epithelium and likely reduces CSF production and glymphatic circulation (Damkier et al., 2013; Benveniste et al., 2017). Recently, Liu et al. also reported that CSF production was increased by a combination of α_1_-, α_2_-, and non-selective β-adrenergic receptor antagonists, as well as some anesthetics, including isoflurane, ketamine, and xylazine (Liu G. et al., 2020).

Perioperative hemodynamic optimization is crucial for improving outcomes after surgery and anesthesia. Anesthesia management often includes the administration of catecholamine vasoactive drugs such as norepinephrine, dopamine, and phenylephrine to maintain stable hemodynamics throughout surgery (Foss and Kehlet, 2019). Norepinephrine, a central regulator, also inhibits glymphatic transport (Jessen et al., 2015). However, Iliff et al. demonstrated that dobutamine increased the pulsatility of the penetrating artery by 60% and enhanced the glymphatic influx (Iliff et al., 2013). Furthermore, catecholamines can modulate astrocyte function and signaling to regulate the glymphatic system (Fuxe et al., 2015). The astrocytic dopamine and adrenergic receptor subtypes are significant drug targets in neurological and psychiatric diseases (Fuxe et al., 2015). Norepinephrine and dobutamine also work together to affect glymphatic function by acting on fluid availability and convective fluxes through various mechanisms. Therefore, catecholamines may be the primary regulators of solute clearance from the brain.

Theoretically, any changes in cardiovascular parameters induced by anesthesia can impair the cardiac pulsation mechanism, resulting in waste accumulation. Hence, understanding the processes contributing to pulsatility and how these components drive waste clearance by the glymphatic system continues to evolve. Further research is needed to determine the most appropriate management of glymphatic function during clinical anesthesia.

### Respiration Changes

During experimental testing in mouse or rat models described above, spontaneous respiration was inhibited but still preserved in research involving the glymphatic system. Spontaneous respiration in anesthetized animals is very different from that in controlled or assisted respiration under clinical anesthesia. During clinical anesthesia, positive-pressure mechanical ventilation is often used to replace spontaneous respiration. Mechanical ventilation differs from spontaneous breathing in terms of physiology, and its effect on glymphatic system transport in the brain is currently unknown. Ventilators are inspiratory assist devices that deliver tidal breath under positive pressure. Positive pressure ventilation, including positive end-expiratory pressure, may increase intrathoracic pressure and reduce venous return, subsequently causing increased ICP (Chen et al., 2018). Positive pressure ventilation also reduces cerebral perfusion pressure due to increased ICP and decreased mean arterial pressure (Chen et al., 2018). It has been hypothesized that ventilation impairs respiration, causing waste accumulation in the brain.

Although the administration of local anesthetics through the spinal or epidural canal can provide anesthesia without the need for mechanical ventilation, there are still additional effects on the sympathetic nervous system, parasympathetic nervous system, and motor control, resulting in respiratory impairment (Wink et al., 2016). Theoretically, these changes impair the function of the glymphatic system. Some respiratory parameters, such as tidal volume, frequency, and airway pressure, have not been studied in relation to glymphatic activity.

Preclinical and clinical evidence suggests that mechanical ventilation induces adverse neurocognitive effects (Wang et al., 2015). It is estimated that 70–100% of critically ill patients have cognitive impairment after mechanical ventilation, and 20% of patients still have cognitive impairment 5 years later (Herridge et al., 2016; Bilotta et al., 2019). In patients with COVID-19, delirium/neurological disorders are clearly correlated with prolonged mechanical ventilation (Alonso-Lana et al., 2020; Helms et al., 2020). However, the mechanism by which mechanical ventilation affects the incidence of postoperative cognitive disorder remains unclear. The discovery of this novel glymphatic pathway may provide clues for future research. Evaluating perioperative glymphatic function might provide new insights for understanding the pathophysiological mechanisms underlying cognitive decline during clinical anesthesia and surgery.

### Arousal Regulation Mechanism Altered by Anesthesia

Some studies have demonstrated that arousal state correlates with the glymphatic system (Hablitz et al., 2019), while the arousal state also reflects “depth of anesthesia” in clinical settings (Shalbaf et al., 2015). The arousal state is closely related to hemodynamic rhythms such as blood pressure, heart rate, and blood oxygenation level-dependent signals, which affect glymphatic function (Fultz et al., 2019). The depth of anesthesia can affect not only the hemodynamics but also the arousal mechanism. Therefore, there may be a relationship between anesthesia depth and glymphatic function (Shalbaf et al., 2015). General anesthesia is characterized by the suppression of central arousal controlled by the locus coeruleus and noradrenergic tone (Brown et al., 2010). Norepinephrine release is reduced when anesthesia suppresses sympathetic nerve activity. It has been confirmed that noradrenergic blockade or reduction produces the same changes in ISF volume fraction in the glymphatic system (Xie L. et al., 2013).

However, accurate estimation of the depth of anesthesia remains an important issue in clinical or animal anesthesia. Non-invasive EEG or evoked potential indices, such as the bispectral index, narcotrend index, state entropy, and response entropy, for monitoring anesthesia depth are widely used in clinical research. High EEG delta power is positively associated with glymphatic activity, while beta power is negatively corelated in animal models (Hablitz et al., 2019). When bispectral index-guided deep anesthesia is used in geriatric anesthesia, postoperative delirium and cognitive decline in elderly surgical patients are reduced (Deschamps et al., 2019; Miao et al., 2019). Therefore, intraoperative indicators for monitoring anesthesia depth are clinically significant for research about the relationship between glymphatic function and PND. Insufficient anesthesia can cause physiological and psychological injuries, whereas overdose anesthesia may induce hemodynamic disturbances. Therefore, monitoring the depth of anesthesia may also prevent impairment of the glymphatic system and cognition due to inadequate or excessive anesthesia.

### Astrocytes and AQP4 Function Changes

The use of *in vivo* two-photon imaging demonstrated that calcium signaling in astrocytes is associated with the selective transport of small lipophilic molecules and rapid ISF movement throughout the glymphatic system (Rangroo Thrane et al., 2013). A recent study found that glymphatic flow is regulated by fluid shear stress produced by perivascular CSF or ISF dynamics, which are capable of mechanically opening NMDA receptors and producing increased Ca^2+^ currents in cultured astrocytes (Maneshi et al., 2017). General anesthetics, especially ketamine, inhibit NMDA receptors and reduce Ca^2+^ signaling (Duman et al., 2019), likely to regulate glymphatic function through this mechanism. The anesthetic sevoflurane induces changes in astrocyte morphogenesis by downregulating ezrin, an actin-binding membrane-bound protein, in addition to disrupting astrocyte Ca^2+^ currents acutely and chronically (Zhou et al., 2019). Moreover, brain surgery such as cisterna magna puncture can cause spontaneous frequent but asynchronous astrocytic Ca^2+^ signaling and impair glymphatic lipid transport, consequently increasing intracellular lipid accumulation (Rangroo Thrane et al., 2013).

AQP4 colocalizes the inwardly rectifying potassium channel Kir4.1 and contributes to the coupled influx of water and K^+^ after neuronal activity (Jo et al., 2015). Accordingly, some anesthetics can impair AQP4 function by blocking potassium channels (Ou et al., 2020), leading to glymphatic function changes. In recent studies, 2.5% sevoflurane has also been shown to increase glymphatic function by upregulating AQP4 expression in astrocytes (Gao et al., 2019). Changes in AQP4 expression and depolarization are functionally relevant to the glymphatic system and cognition (Hubbard et al., 2018).

## Glymphatic Function Affected by Surgery

Persistent systemic inflammation caused by surgery can disrupt the integrity of the BBB (Vacas et al., 2014; Hughes et al., 2016; Bi et al., 2017; Wang et al., 2017; Ni et al., 2018; Verheggen et al., 2018). The BBB is a complex functional and anatomical system that prevents the entry of neurotoxic plasma elements, blood cells, and pathogens into the brain. It is comprised of endothelial cells, perivascular microglia, pericytes, neurons, and astrocytic end-feet (Sweeney et al., 2019). Tight junction proteins located between adjacent endothelial cells include claudins, tricellulin, occludin, and zonula occludens (Sweeney et al., 2019). Acute endothelial dysfunction caused by surgically induced systemic inflammation may lead to BBB disorders (Plog et al., 2015; Ekeloef et al., 2017). Inflammation after laparotomy can decrease the levels of tight junction proteins such as claudin, β-catenin, occludins, and ZO-1, all of which can contribute to tight junctions and BBB permeability (Yang et al., 2017). Moreover, astrocytes are easily activated by postoperative inflammatory cytokines such as TNF-α, IL-1β, IL-6, complement C3, and high mobility group box-1, which can subsequently damage the integrity of the BBB, especially in the hippocampus (He et al., 2012; Jin et al., 2014; Xiong et al., 2018). Activated astrocytes can also encourage tissue proliferation, resulting in scar-like perivascular barriers (Voskuhl et al., 2009). The endothelial cell membrane and the BBB close junctions comprise the vascular components of the glymphatic system, while the avascular components consist of astrocyte end-feet integrated with glia-line boundaries between the neuropil and axon tract regions (Plog and Nedergaard, 2018). Therefore, systemic inflammation influences the function of the glymphatic system based on the evidence that the structure and function of the glymphatic system are closely related to that of the BBB (Verheggen et al., 2018). Notably, a recent study found that lipopolysaccharide-induced systemic inflammation reduced perivascular CSF tracer flow and penetration into the parenchyma (Manouchehrian et al., 2021). These observations may be beneficial for our understanding of the role of systemic inflammation in glymphatic clearance.

### How the Surgical Position Influences Glymphatic Transport

Researchers have studied the effects of body position on glymphatic function and Aβ clearance using optical imaging, CSF radiotracers, and fluorescent tracers in the brains of anesthetized rodents. According to previous studies, glymphatic transport is most effective in the lateral position, inferior in the supine position, and least efficient in the prone position (Lee et al., 2015). Thus, it is clear that postural or gravitational factors also exert regulatory control over the glymphatic pathway. Thus, we speculate that in postures with the head down, minor changes in CSF pressure, such as ICP and hydrostatic pressure, increase due to gravity; tissue pressure or vascular function could alter the shape of perivascular spaces and accelerate glymphatic drainage problems, impairing cognitive function.

Although PND was initially described as a complication after cardiac surgery, many recent studies have found that some patients undergoing non-cardiac surgeries, such as orthopedic surgery, also suffered cognitive decline (Evered and Silbert, 2018). Due to the various types of surgery, surgical posture may be an essential but overlooked factor in these studies. Some prone surgeries, particularly spine surgery, may cause postoperative cognitive impairment (Kim et al., 2016; Ezhevskaya et al., 2019). Furthermore, Trendelenburg, a supine position used in laparoscopic surgery in which the patient's head is lower than the feet, can result in postoperative cognitive decline. This position could increase intra-abdominal pressure, reduce cerebral venous outflow, and impair lymphatic function, similar to positive pressure ventilation or positive end-expiratory pressure (Maerz et al., 2017). Some studies have even demonstrated that sleeping in the supine position has been shown to reduce cognitive function in healthy volunteers, while sleeping in the upright position did not affect the participants (Muehlhan et al., 2014). According to studies on posture and glymphatic function, the surgical position can worsen the cognitive impairment, especially in prolonged surgeries, including cardiac surgery. Understanding the effects of different body postures on the glymphatic clearance pathway may be necessary for interpreting the procedure of PND.

### Perioperative Sleep Disorder Leads to Glymphatic Dysfunction

The clearance of the glymphatic system is closely related to the sleep-wake cycle, with sleep promoting faster metabolite removal (Xie L. et al., 2013). However, perioperative sleep is frequently disrupted by many factors, including postoperative pain, environmental and surgical stress, anesthesia, and other factors that lead to discomfort (Whitlock et al., 2017; Su and Wang, 2018). Postoperative sleep disruptions manifest as sleep fragmentation and reduced slow-wave and REM sleep durations (Chouchou et al., 2014). In particular, REM sleep disorder may significantly negatively affect postoperative cognition (Lazic et al., 2017). Subsequently, the glymphatic system is impaired and degrades with sleep disturbances (Nedergaard and Goldman, 2020). After a night of sleep deprivation, protein Aβ levels in the thalamus and medial temporal structures are also elevated in healthy individuals (Shokri-Kojori et al., 2018). Moreover, sleep disruption increases the neuronal activity and generates more waste products, including lactate, which are exported via glymphatic fluid transport (Lundgaard et al., 2017).

Postoperative pain remains a significant health care issue that disturbs sleep in the postoperative period, and sleep disturbances may, in turn, exacerbate postoperative pain (Chouchou et al., 2014). Anesthesia is substantially different from natural sleep, sometimes interfering with the circadian rhythm and disrupting the sleep cycle, subsequently affecting glymphatic clearance (Lazic et al., 2017). Additionally, surgical and environmental stress appears to be a significant cause of sleep disruptions during peri-operation (Xu et al., 2016), and chronic stress via glucocorticoid signaling impairs AQP4-mediated glymphatic transport (Wei et al., 2019). This evidence suggests that cognitive decline in patients with sleep disorders may be caused by decreased waste removal and increased waste accumulation in the brain due to glymphatic dysfunction.

Currently, in animal models of glymphatic function, sleep is simulated by anesthesia or sleep deprivation. Owing to technical limitations, it is difficult to perform such experiments on naturally sleeping mice (Xie L. et al., 2013). According to some studies, the effects of sleep and general anesthesia on the glymphatic system are equivalent (Xie L. et al., 2013; Benveniste et al., 2019a). However, anesthesia and natural sleep outcomes remain markedly different; anesthesia can affect hemodynamics, interfere with the biological clock, and disrupt the sleep cycle (Lazic et al., 2017). Thus, the impact of anesthesia on the glymphatic system needs to be investigated further.

## Why Some Patients Are Susceptible to PND

Notably, the adverse cerebral effects of anesthesia were primarily reported in the elderly (Bedford, 1955; Berger et al., 2015). It is not yet fully understood why elderly patients are highly susceptible to PND, while young people who undergo surgery rarely develop it (Hu et al., 2020). Researchers have discovered some PND-susceptible patients with preoperative disease, such as preclinical AD (Xu et al., 2014), hyperglycemia (Zhang et al., 2014), cerebral microemboli (Wang et al., 2020), and other underlying neurovascular diseases or neurodegeneration (Wang et al., 2020). These preoperative diseases may share common underlying mechanisms and contribute to neurocognitive dysfunction or dementia. With advances in imaging technology, it has been found that these diseases have glymphatic impairment (Gaberel et al., 2014; Kress et al., 2014; Kyrtsos and Baras, 2015; Jiang et al., 2017; Hadjihambi et al., 2019). A theory states that susceptible patients with PND and aging have preexisting glymphatic dysfunction but progresses slowly without significant cognitive impairment (Bolte et al., 2020). Perioperative risk factors exacerbate the preexisting glymphatic damage beyond the compensatory limit, resulting in significant cognitive changes in these individuals. AD is a progressive neurodegenerative disorder characterized by gradual cognitive decline, which is characteristic of extracellular Aβ deposition and intracellular accumulation of hyperphosphorylated tau (Long and Holtzman, 2019). Patients in the preclinical stage of AD, particularly those with APOE4 genetic risk factors (Mentis et al., 2020), already have Aβ accumulation and impaired glymphatic clearance, but no clinically detectable symptoms of cognitive impairment (Masters et al., 2015). Moreover, aging is associated with degenerative changes in the brain's structure and function, such as reduced cerebrovascular pulsatility, depolarized or downregulated AQP4, and sleep deprivation, which impair glymphatic function (Zeppenfeld et al., 2017). Glymphatic dysfunction may result in the deposition of toxic solutes (including amyloid) in the aging brain, which exacerbates glymphatic dysfunction and creates a vicious cycle (Iliff et al., 2013). Vascular dementia is caused by many neurovascular diseases, including hypertension, ischemic stroke, traumatic brain injury, and microinfarcts, which significantly impair arterial CSF pulsation and glymphatic clearance (Riba-Llena et al., 2018). Diabetes can also impair cardiac pulsation due to pathological changes in the vascular endothelium (Jiang et al., 2017).

Due to technical limitations, few previous studies have used non-invasive methods to investigate glymphatic function in humans, particularly hospitalized surgical patients susceptible to PND. Recent advances in imaging, such as real-time MRI (Ding et al., 2018), ultra-fast MRI (Kiviniemi et al., 2016), and gadolinium-based contrast agents enhanced MRI (Ringstad et al., 2018; Deike-Hofmann et al., 2019), will provide a sensitive and non-invasive tool for assessing glymphatic function in clinical trials. Notably, it is essential to develop intraoperative monitoring tools for glymphatic function. A new method of measuring human glymphatic function that uses near-infrared spectroscopy has been developed for long-term monitoring of brain function, which is highly compatible with perioperative monitoring (Myllylä et al., 2018). These non-invasive methods may help translate glymphatic measurements from the laboratory to the clinic (Jiang, 2019). Advances in technology will yield new and valuable information about the glymphatic system, providing a quantitative map for the diagnosis, monitoring, and prognosis of neurological disorders, including PND.

## Conclusion and Perspective

Recent discoveries concerning the glymphatic system anatomy and mechanisms have contributed to a better understanding of CSF circulation and waste removal. Furthermore, the glymphatic system has an immune function and may affect the onset and progression of PND, either directly or indirectly. Because clinical research on perioperative glymphatic changes is still lacking, scientists and clinicians, particularly gerontologists, neurologists, psychiatrists, anesthesiologists, and surgeons, can collaborate to uncover the etiological mechanisms of perioperative glymphatic changes. The glymphatic concept may provide a strategic breakthrough in rethinking, treating, and, most importantly, preventing PND.

Human studies exploring the physiological and pathological mechanisms between the glymphatic system and brain cognition over time have greatly improved our knowledge; however, some key issues still need to be addressed by future research. First, the definition of normal or healthy glymphatic function may be one of the biggest challenges. A simpler and quicker intraoperative monitoring method remains to be developed. A better description of glymphatic dynamics will help demarcate “normal” and “abnormal” glymphatic function in humans and identify preventive strategies for PND. Second, perioperative factors such as anesthetic type, intraoperative hemodynamics, surgery position, surgical inflammation, and previous health status may confound research on the glymphatic system and PND. Cross-sectional clinical studies have demonstrated specific changes in the glymphatic transport of patients susceptible to neurological diseases, which is helpful in predicting the incidence of PND. Third, more clinical studies should be conducted instead of purely animal studies. Although association analysis may provide critical information for cause-effect deductions, correlation does not necessarily mean causation. Fourth, basic research targeting the perioperative glymphatic system in PND merits optimization; specifically, appropriate model systems should be carefully selected. Finally, the translation of basic research results into clinically relevant effects in humans should be expedited. Most data on the role of the glymphatic system are based on animal studies. Preclinical animal studies frequently result in unexpected failures during clinical transition due to unidentified reasons. Alternatively, future therapeutic interventions will likely be based on individual factors due to significant differences in anatomical variation and disease complexity among human populations.

## Author Contributions

XR, GZ, LL, and CL contributed significantly to conception of the review and edited the review. XR undertook the literature, drafted, and corrected most of the manuscript and tables. SL, KL, and HL assisted with part of the pictures and manuscript. CL contributed to the sections of glymphatic system and anaesthetics. All authors contributed to the article and approved the submitted version.

## Conflict of Interest

The authors declare that the research was conducted in the absence of any commercial or financial relationships that could be construed as a potential conflict of interest.
